# Patients’ and Caregivers’ Conceptualisations of Pressure Ulcers and the Process of Decision-Making in the Context of Home Care

**DOI:** 10.3390/ijerph16152719

**Published:** 2019-07-30

**Authors:** Francisco José García-Sánchez, Vicente Martínez-Vizcaíno, Beatriz Rodríguez-Martín

**Affiliations:** 1 Department of Nursing, Physiotherapy and Occupational Therapy, Faculty of Nursing, University of Castilla-La Mancha, 13071 Ciudad Real, Spain; 2 Social and Health Research Center, University of Castilla-La Mancha, 16327 Cuenca, Spain,; 3 Faculty of Health Sciences, Universidad Autónoma de Chile, Santiago 3467987, Chile; 4 Department of Nursing, Physiotherapy and Occupational Therapy, Faculty of Occupational Therapy, Speech Therapy and Nursing, University of Castilla-La Mancha, Av. Real Fábrica de Sedas s/n, 45600 Talavera de la Reina (Toledo), Spain

**Keywords:** shared decision-making, pressure ulcer, caregivers, home nursing, qualitative research, grounded theory

## Abstract

*Background*: Although the addition of patients in the process of shared decision-making can improve their recovery, there is a lack of knowledge about patients’ and caregivers’ perceptions on the management of pressure ulcers at home. *Objectives*: To explore the conceptualisations of patients with pressure ulcers and their caregivers on the barriers and facilitators for their involvement in home care and in the process of shared decision-making regarding the care provided. *Methods*: A qualitative study based on grounded theory in a theoretical sample of 10 patients with pressure ulcers and 15 main caregivers from the health district of Puertollano (Spain). The data were based on semi-structured interviews, analysed using a coding process and the constant comparative method. Results: According to the participants, personal motivation and the involvement of primary care professionals facilitated their participation in the process of shared decision-making and generated feelings of positivity. In contrast, older age, having disabling pathologies, a low educational level or health paternalism were perceived as barriers for their involvement. *Conclusions*: A non-paternalistic care model and personal motivation facilitate the process of shared decision-making in the care of people with pressure ulcers. Further studies are required to deepen the understanding of this phenomenon and examine the barriers and facilitators for the involvement of patients and caregivers in the management of these injuries in other contexts.

## 1. Introduction

Recent studies recommend that health care should be evidence based, while encouraging patient-centred decision-making [1,2]. Thus, over recent years, the paradigm of shared decision-making (SDM) has emerged. This paradigm incorporates the patients’ values and preferences with regard to a health problem after being informed by health professionals of the possible therapeutic alternatives and anticipated results [3,4], therefore providing patients with an active role in decision-making and the planning of care [5]. In order to apply SDM, it is necessary to respect the patient’s autonomy, abandoning paternalistic models regarding the relationship between health professionals and patients/family members and respecting the principle of patient autonomy [5,6,7].

A recent Cochrane review affirms that patients who are better informed also perceive the risks associated with their pathology better and acquire a more active role in the process of SDM about their health [4]. Several studies and scientific organisations suggest that the combined effort of professionals and patients, when based on evidence and supported by systems that promote decision-making, improves the results of chronic illness care [8,9,10]. Shared decision-making empowers patients and helps them to improve the self-management of their disease; this self-control of their pathology is essential for daily decisions that positively influence the course of the disease [10,11,12,13].

Patients with chronic wounds and their caregivers do not feel involved in the decision processes regarding the prevention and care of their wounds. A communication system between patients, caregivers and health teams should be developed to enable informed evidence-based decisions about the person’s health care, in such a way that the patients and caregivers’ voices are taken into account when assessing the value of health care options [14].

Despite the knowledge that patient involvement in care can be an effective pressure injury prevention strategy, and that a majority of patients prefer to take a proactive role in pressure injury prevention, barriers have been identified that made it difficult for them to participate in the prevention and care of these chronic wounds [15]. Considering the above, it is essential to understand what are the tools that facilitate the process of SDM, as well as the barriers for its implementation. This information could be useful for the development of clinical practice protocols and guidelines that promote patient-centred care [16,17,18,19].

It is known that pressure ulcers (PUs) are a major public health problem that increase morbimortality, the amount of care needed and the lack of autonomy or dependency of patients [15]. The home is perceived as the preferred place for the care of people with PU [20,21,22], with the main caregiver (MC) acquiring an essential role in patient care and the resolution of the PU [22].

Prior research states the need of including the point of view of both people with PU and their caregivers and of including their treatment preferences in order to improve the management of PUs [15,23,24]. Despite this, to the best of our knowledge, no studies to date have examined the conceptualisations of people with PUs and their caregivers on their participation in the process of SDM in home care.

We aim to explore the conceptualisations of patients with pressure ulcers and their caregivers on the barriers and facilitators for their involvement in home care and in the process of shared decision-making regarding the care provided.

## 2. Materials and Methods

This study is part of a line of research on the home care of pressure ulcers from the point of view of patients and their caregivers, investigating their conceptualisations of the care, barriers and facilitators with relation to being involved in the same and shared decision-making.

A study was designed, aimed at examining the conceptualisations of patients and caregivers regarding the home care of pressure ulcers, together with the barriers and facilitators for their involvement in home care and shared decision-making in this pathology.

### 2.1. Design

A qualitative study was designed, based on grounded theory in a theoretical sample of patients with PU and main caregivers of people with PU [25,26,27]. As a data collection technique, we used semi-structured interviews with a theoretical sample of 10 people who had a PU, either currently or in the past, and 15 caregivers with a current or past experience of caring for the same. This inductive method was used as it provided a theoretical explanation of the perception of participants of the process of SDM in the home care of PU.

### 2.2. Sample and Data Collection

A theoretical sample was used with the following inclusion and exclusion criteria. In the case of people with PU, the inclusion criteria were: 1) people over the age of 18 of both sexes and who had received treatment over at least 30 days, between the years 2014 and 2015, due to a PU of category II, III or IV, according to the European Pressure Ulcer Advisory Panel Classification (EPUAP) [28,29], and 2) people belonging to one of the basic healthcare districts of Puertollano (Ciudad Real). Candidates were excluded from participation if they had acute injuries, a diagnosis of cognitive decline or mental pathology, or were unable to communicate in Spanish. For the main caregivers, the inclusion criteria were: 1) people over the age of 18, who were the main informal caregivers of people with PU, and 2) not perceiving a financial remuneration for care. Likewise, we excluded those caregivers who were unable to communicate in Spanish, and those who had a cognitive decline or another pathology that hampered their participation in the interview.

To ensure the participation of patients and caregivers of both sexes, different ages and varying sociodemographic characteristics (civil status, level of studies, category and location of PU, place where PU developed, days of evolution, pain, kinship of caregivers and work situation), a theoretical sampling was performed to the point of data saturation, at which point expanding the sample would not lead to the emergence of new concepts [26,27]. Table 1, Table 2 and Table 3 feature the main characteristics of the study participants.

The interviewer used two theme guides, one for the patients and one for the caregivers. These included the main respective themes, which were duly refined during the interview process (Table 4 and Table 5).

All the interviews were conducted in the homes of the participants during the years 2015 and 2016. The interviews lasted between 45 and 60 minutes and were digitally audio recorded after obtaining the consent of the participants.

### 2.3. Ethical Issues

The study was approved by the Clinical Research Ethics Committee (CEIC) of the General University Hospital of Ciudad Real (Spain) (reference 11/2014). We also requested the authorisation of the directors of the participating health centres, and the data management process was in accordance with the Helsinki Declaration and the personal data protection regulations of Spanish and European laws. All participants signed to give informed consent after receiving a comprehensive explanation of the study.

### 2.4. Data Analysis

All interviews were anonymised before verbatims were transcribed, in order to guarantee the confidentiality of the treatment of the data. After this, the transcriptions were analysed to discern the conceptualisations of the participants of the process of SDM in the home care of PUs and to obtain a theoretical explanation of the phenomenon. The analysis of the transcriptions was performed independently by two researchers who were experts in qualitative methods. Thereafter, the final results were agreed upon by consensus. Any discrepancies were resolved by a third researcher [30,31,32].

Following the principles of grounded theory, we used the method of constant comparison. This method entailed the continuous review and comparison of the data, in order to elaborate and compare novel study categories and seek a theory to explain the study phenomenon from the participants’ point of view [27]. The researchers shared operational memos with the emerging codes and agreed on the categories and subcategories. Accordingly, a new hermeneutic unit for the project was created and shared by the researchers. Additional examples of operational memos, codes, categories and subcategories are showed in the supplementary Table S1.

Thus, constant advances and regressions took place between the interview transcriptions, the theoretical ideas behind the codes, their transcriptions and the literature review [30]. Theorizing was a part of the data analysis process [33,34], together with open, axial and selective coding [26,27]. During the data analysis, a theory was developed to explain the study phenomenon from the participants’ point of view [27,31,32]. The open coding process enabled us to fragment the data into small meaningful units, which were organized by categories. We were able, via a process of axial coding, to relate the categories and subcategories. Lastly, the selective coding process enabled us to elaborate a theory to explain the relationships by integrating the categories based on a central key explanation.

During the analysis and coding of the data, we used the ATLAS-Ti 7.5.13 computer program (ATLAS.ti Scientific Software Development GmbH, Berlin, Germany). 

### 2.5. Validity

The transcriptions were returned to the participants to verify the interview contents. Beside the literal transcription of the interviews, the use of the method of constant comparisons and the triangulation methods contributed toward the reliability and validity of the conclusions and their content. Two researchers, experts on qualitative methods, analyzed and triangulated the data to guarantee the reliability and validity of the conclusions. Any disagreements were referred to a third reviewer. Furthermore, as people with different characteristics were included, data triangulation was performed according to the study phenomenon by including a theoretical sample of both caregivers and people with PU, all of whom were of a different sex, age, and with varying clinical and sociodemographic characteristics [34].

## 3. Results

After a data analysis, two barriers (personal limitations and organizational characteristics of the health system) and two facilitators (personal motivation and the involvement of primary care professionals) emerged in the process of making shared decisions in the home care of pressure ulcers. Within the facilitators, the code “The involvement of primary care professionals” resulted in the emergence of three categories: “Closeness”, “Trust” and “The attitude of professionals regarding their open mindedness to the opinions of patients and family members”, including two subcategories in this last category: “Effective communication” and “The need to be listened to”. The other facilitator’s code, “Personal motivation”, resulted in the following category: “Positive feelings associated with the implication in decision-making”, and four subcategories: “Wellbeing”, “Peace of mind”, “Feelings of productivity” and “Willingness to participate in the drafting of clinical practice guidelines”. Within the barriers, in the code “Limitations related to participants’ personal factors”, three categories appear: “Advanced age”, “Disabling pathology” and “Low educational level”. In the code “Limitations related to the organization of the health system”, one category appears: “Influence of the paternalistic model of organization of care”.

**Figure 1:**
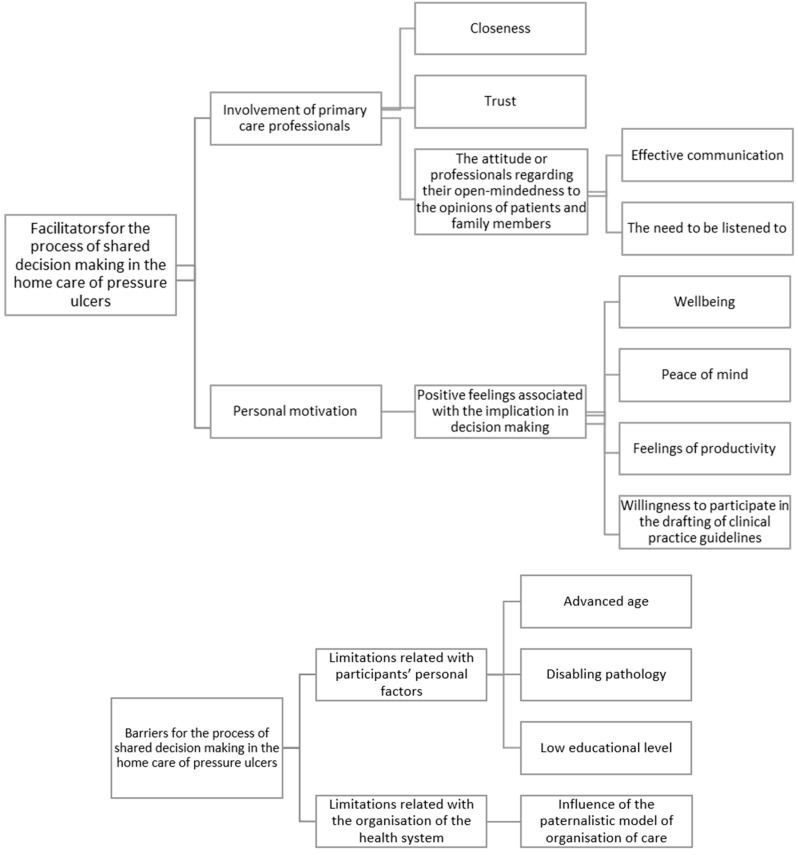
Facilitators and barriers in the process of shared decision-making in the home care of pressure ulcers.

Figure 1 displays the code diagrams, illustrating the relationship between the main categories and the codes found. Furthermore, to facilitate the understanding of the results, a selection of the participants’ most representative quotes are included.

### 3.1. Facilitating Elements for the Process of Shared Decision Making in the Home Care of Pressure Ulcers

#### 3.1.1. Involvement of Primary Care Professionals

The involvement of primary care professionals, especially nursing professionals, facilitated the participation of both people with PU and their caregivers in the process of SDM. The following factors were perceived by participants as being facilitating elements for the involvement of primary care professionals: closeness, trust and an attitude of being open to the opinions of patients and family members.

#### 3.1.2. Closeness and Trust

The participants described that the environment of proximity and trust that the primary care professionals engendered when they went to their homes contributed toward their involvement in care. They highlighted the fact that health professionals were the professionals who contributed the most to the creation of such an environment.

“With the nurse at the health centre there is more trust, just as with the doctor, and the relationship is closer, they talk to you and they ask you a lot about how you are and whether what they put on you bothers you and what you prefer them to use” (P.2).

“I talk a lot with my nurse, he’s nice and I have a very good relationship. I told him when it hurt the most and when I was better, he saw how I was improving or worsening, as at times I got worse” (P.7).

“Very good, with the nurse who comes to the house it was great, he does it all very well and we really trust him. He asked for my opinion on how to treat me and on what I felt was the best way to do it.” (P.8).

#### 3.1.3. The Attitude of Professionals Regarding Their Open-Mindedness to the Opinions of Patients and Family Members

The participants valued being listened to by the professionals, demanding their right to voice their opinion and for their opinions to be taken into consideration, especially when they could share their experience as caregivers. All these aspects were considered by participants as being essential for attaining an effective communication between the patient/family member and the professional.

“Whenever I communicated well with them (doctor and nurse) and it was good; it gave me a feeling of trust”. (CP.5).

“He explains everything to me (the nurse) and he asks me what I think is best. It is very important to know for those of us who don’t know. It’s good that the patients are listened to and that we can voice our opinion”. (P.10).

“We know something due to the experience of caring and I believe it wouldn’t be bad to be asked and we could say something” (CP.10).

#### 3.1.4. Personal Motivation

The participants considered that their own personal motivation facilitated their involvement in the process of decision making, associating this motivation with the emergence of positive emotions. In addition, certain participants expressed their willingness to participate in the write-up of technical documents on the care of people with PU.

#### 3.1.5. Positive Feelings Associated with Involvement in the Process of Decision Making

The opportunity for participating in the care and collaborating in decision-making was associated with the appearance of the following positive feelings in participants: wellbeing, peace of mind, the feeling of being productive and the willingness to participate in the drafting of technical documents.

#### 3.1.6. Wellbeing

“They asked me and I said what I thought, because I wanted her to get better and not suffer. There was an exchange of opinions between the doctor and the nurse with myself and that made me feel good” (CP.1).

#### 3.1.7. Peace of Mind

“At home, I was always present during the care of the wound and I helped the nurse during the task. I told the nurse about the progression and how I saw my brother. I like this way of working more, it gives me greater peace of mind” (CP.6).

#### 3.1.8. Feelings of Productivity

“At home, I helped with the care for the wound and that made me feel good as I was helping to cure my father and that way I also saw how it was going” (CP.5).

“The truth is that you feel better if you participate and you see how it evolves and if it gets better you feel that your care gives results and you feel happy” (CP.10).

#### 3.1.9. Willingness to Participate in the Drafting of Clinical Practice Guidelines

The development of clinical practice guidelines involves incorporating the opinion and experience of patients and caregivers on the prevention and treatment of several pathologies [35,36,37,38], as an adjuvant part of the technical tools that professionals have for dealing with these chronic diseases.

The participants considered that they had to respect the specialised knowledge of healthcare professionals, but also expressed their desire to participate in the elaboration of clinical practice guidelines on the prevention and the care of PU, perceiving that this could increase their personal experience of caring for a person with PU:

“We can give our opinion, it’s not that we know much, but it is important for them to consider you”. (CP. 11).

“However, if there are people who know, well of course you can hear what they say, but always respecting what doctors and nurses say, as these are the people who know more about this because it is what they do”. (CP.15).

Some participants considered that their perceptions and experiences should be incorporated into guidelines or technical documents, as these would provide an added value to care:

“We are the patient’s side and we can provide information that helps to cure the person better, and we also see the evolution, I guess that could be taken advantage of”. (CP.10).

“It’s good that the patients say things, especially how to treat patients, how to treat wounds, I think that the person who knows most about this are those who have studied and those of us who don’t know anything, well we have to let them do things to us, but, yes, it’s good that we talk” (P.7).

### 3.2. Barriers for the Process of Shared Decision Making in the Home Care of People with Pressure Ulcers

#### 3.2.1. Limitations Related to Participants’ Personal Factors

The participants considered that being of an advanced age, having a disabling pathology or having a low educational level were barriers for their involvement in the process of SDM in the home care of PUs.

#### 3.2.2. Advanced Age

Being of an advanced age was considered by participants as being a barrier for their participation in care:

“I am 93 years old and I almost can’t see. I am old and I am ill. I don’t do anything because I simply can’t, not because I don’t want to”. (P.4.)

#### 3.2.3. Disabling Pathology

On other occasions, the participants acknowledged that, besides having PUs, suffering from other concomitant pathologies such as infections or postsurgical convalescence hampered their implication in the process of SDM.

“I used to have fever and I felt bad, but what I experienced that day is a very bad memory. I didn’t feel well; I was fed up with everything. I could not help with the care of the wound. I felt very dependent, but I couldn’t do anything”. (P.2).

#### 3.2.4. Low Educational Level

For some participants, their low educational level was a barrier for being involved in the care of PUs. 

“Oh, I don’t know anything, I can’t say much, except for doing what they tell me to do. Here those who know are the doctors and nurses, they are the ones who have studied”. (CP. 12).

“I don’t know. The ones who know the most about this are the doctors and nurse who have studied, I hardly know how to read and write, what am I going to say”. (CP.15).

#### 3.2.5. Influence of the Paternalistic Model of Organisation of Care

Some participants highlighted that the influence of the paternalistic model in biomedical care hampered their participation in the treatment of their wounds. In these cases, the participants perceived that they were patients or family members who lacked the opportunity to make decisions, as all the decision-making was made by the health professionals.

“For some professionals, even if I were a nurse, I was a patient with a pathology and the professional was the professional. You had neither a voice nor a vote, nor did your opinion count, which is another thing that I hope one day we can change. At that moment in time, the person becomes a patient who has to abide by whatever the professional says”. (P.1).

“At home, depending on who came, they let you help with the care for the wound and help out, but some nurses organised things differently, they did everything and they didn’t let me participate. I prefer helping, as I can see how she is getting on and whether she improves”. (CP.14).

## 4. Discussion

Following the line of a previous study, this study confirms that proximity, trust and an effective and bidirectional communication between people with PU/caregivers and health professionals are essential aspects for the effective participation of patients and their family members in the process of SDM [5]. Furthermore, the contributions of this study are that, in the case of care administrated by primary care, nursing professionals are those who most actively promote the involvement of patients and their family members in the process of SDM. This result has already been found in other care contexts [7,9,10]. Additionally, as noted, the results of this study show that the effective participation of the patient in the process of care requires the existence of an appropriate relationship between nursing professionals and patients [15,39].

Our findings are consistent with previous research, suggesting that not all patients are willing to participate in decision-making concerning their care [7,39,40]. Despite this, the participants felt that professionals should listen to them, provide more information on the care process and request their opinions and experience regarding their care. Moreover, our results agree with those from previous studies that highlight the need for patients and caregivers to receive further information on their health status and the available treatment alternatives [41,42], as well as having a more active role in decisions related to their care process [43,44]. In addition, the results of this study confirm the tendency for a decreased resistance of patients to participate in the process of SDM [4].

Furthermore, as reported in prior studies, the caregivers described how caring for their family member was associated with positive feelings, such as wellbeing or satisfaction [20,45], which contributes toward creating a positive and pleasant care environment and, in turn, promotes the involvement of caregivers in the process of SDM. This process is also perceived as being a psychological strategy toward facing the illness [46], helping to decrease the caregiver’s burden [47,48].

The results of this study highlight the availability and interest of people with PU and their caregivers to collaborate as much as possible in the drafting of guidelines or technical documents on the care of people with PUs. This confirms the need to incorporate the points of view of patients/caregivers in strategies for the prevention and treatment of PUs [15,19]. At present, there is a growing recognition that both patients and family members are part of a process of health decision-making, rather than this being the exclusive prerogative of health professionals [3,4]. To establish the effective participation of patients/caregivers, it is necessary for them to receive information and care that is suited to their knowledge [49].

Moreover, this research shows that having a low educational level can affect patients’/caregivers’ active participation in the process of SDM [40,50]. Therefore, it is especially important to train patients and their caregivers in competencies related to the management of health [20].

Paternalism in health care, perceived by participants as a barrier to their involvement in SDM, follows the findings of previous studies that highlight the need to avoid this type of relationship in the health sector [5,6,7,40].

This research provides a novel point of view, as to our knowledge this is the only study to explore the perceptions of people with PU and their caregivers on SDM in the home care of PU, without including other chronic wounds. This study identified the barriers and facilitators for the involvement of the same in the home care of PU. Nonetheless, further studies are necessary to examine in greater depth the elements that facilitate or hamper the involvement of patients and family members in the process of SDM [7]. Moreover, it would be interesting to analyse this phenomenon in other countries with different care contexts.

### Strengths and Limitations of the Study

The use of qualitative methods does not allow for a generalisation of the findings; however, this study has contributed toward understanding the in-depth perceptions of the main caregivers of people with PUs, thus providing new strategies for improving their care [51].

The participants had variable characteristics, which enabled the sample size to be sufficient for data saturation to occur [34].

Due to the lack of similar studies in our field, this research was not influenced by previous studies. Grounded theory and the constant comparative method enabled the possibility of maintaining the theoretical sensitivity of each phase of the study, while enabling the generation of theories based on the data and avoiding preconceived ideas from other studies or previously existing theories [26].

## 5. Conclusions

Our data are important for the home care management of PU, since they highlight that primary care nurses are perceived as the professionals who most actively promote the participation of patients and caregivers in the process of SDM in these injuries. Providing opportunities for participating in the process of SDM regarding the home care of PUs results in feelings of wellbeing in people with PUs, as well as in their caregivers.

The willingness of patients and caregivers to follow guidelines and technical documents on the prevention and treatment of PUs provides an excellent opportunity for managers and health professionals to incorporate the vision of patients and their caregivers. Their support can help ensure a comprehensive approach to this pathology and facilitate SDM.

SDM requires important adaptations and changes to the health system and its professionals to promote the active participation of patients and caregivers, granting them with a voice and taking advantage of the motivation and experience they have in the care of this chronic disease. Effective mechanisms of participation must be strengthened, avoiding paternalism and strengthening fundamental values of primary health care, such as proximity or trust, which generate the security of being heard and taken into account in patients concerning their care, as well as in caregivers.

To avoid healthcare paternalism (considered as a barrier for the involvement of people with PU and their caregivers in SDM), interventions should be introduced that are aimed at training healthcare professionals in more cooperative models of care with patients.

Further studies are needed to analyse this phenomenon in greater depth. This research should take place within different cultures and healthcare contexts in order to confirm the factors that may influence the preferences of patients and caregivers with regards to their participation in SDM.

## Figures and Tables

**Table 1:** Characteristics of the study participants.

P/CP	Sex	Age	Pressure Ulcer (PU) Category	Location of PU	Evolution of PU in Days
P1	Female	48	3	Heel	365
P2	Female	60	2	Sacrum	180
P3	Male	74	2	Heel	270
P4	Male	93	2	Toes	90
P5	Male	63	4	Heel	120
P6	Female	88	2	Sacrum	180
P7	Female	75	2	Other	120
P8	Female	90	3	Heel	450
P9	Female	81	2	Sacrum	90
P10	Male	85	2	Heel	90
CP1	Female	57	2	Heel	450
CP2	Female	28	3	Sacrum	180
CP3	Female	74	4	Sacrum	450
CP4	Female	70	3	Sacrum	545
CP5	Female	35	2	Trochanter	180
CP6	Female	68	2	Heel	270
CP7	Female	89	3	Toes	90
CP8	Female	23	2	Trochanter	730
CP9	Female	63	2	Heel	120
CP10	Male	68	3	Sacrum	730
CP11	Male	55	3	Sacrum	180
CP12	Female	65	4	Sacrum	90
CP13	Male	82	2	Trocanter	365
CP14	Male	87	4	Sacrum	90
CP15	Male	85	3	Sacrum	550

**Table 2:** Principal characteristics of participants with pressure ulcers (PUs).

Variables		Men	Women
Age	45–65 years	1	2
	66–85 years	2	2
	>86 years	1	2
Civil status	Single	1	0
	Married	3	4
	Widow	0	2
Level of studies	Primary studies	4	5
	University studies	0	1
Pressure ulcer (PU) category according to the European Pressure Ulcer Advisory Panel (EPUAP) classification	2	2	4
	3	0	2
	4	2	0
Location of PU	Sacrum	0	3
	Heels	3	2
	Toes	1	0
	Other sites	0	1
Place where PU developed	Hospital	1	3
	Home	3	3
Evolution of PU in days	≤90 days	2	1
	91–180 days	1	3
	181–365 days	1	1
	>365 days	0	1
Pain according to the numerical pain rating scale *	7	1	2
	8	1	2
	9	0	2
	10	2	0

* Pain was assessed using the Numerical Pain Rating Scale from 0–10, where 0 represents the absence of pain and 10 represents the greatest intensity of pain.

**Table 3:** Principal characteristics of the main caregivers of people with pressure ulcers.

Variables		Men	Women
Age of caregivers	25–45 years	0	3
	46–65 years	1	3
	66–85 years	4	3
	>85 years	0	1
Civil status of caregiver	Single	1	3
	Married	1	4
	Widow	3	3
Relationship with the patient	Spouse	3	3
	Son/daughter	2	4
	Grandchild	0	1
	Sibling	0	1
	Neighbour	0	1
Caregiver’s level of studies	Primary education	5	7
	Secondary education	0	1
	University studies	0	2
Caregiver’s work status	Unemployed	1	1
	Housework	0	2
	Employed outside the home	0	3
	Retired	4	4

**Table 4:** Interview guide used with people with pressure ulcers.

Experiences related to the pressure ulcer (PU): personal meanings and changes in daily life after the appearance of a PU. Relationship with informal caregivers after the appearance of a PU. Perception on the care received by the professionals who treat PUs. Preferences and areas of improvement for the treatment of PUs. Willingness to participate in shared decision-making.

**Table 5:** Interview guide used with the main caregivers of people with pressure ulcers.

Personal experience of the care of a person with pressure ulcers (PUs): personal experience of care, assessment of the care administrated to a person with a PU, concerns and fears regarding care, changes in daily life and changes in family dynamics. Perception of the care received on behalf of professionals who treat PUs. Preferences and areas of improvement concerning the treatment of PUs. Willingness to participate in shared decision-making.

## Supplementary Materials

The following are available online at https://www.mdpi.com/1660-4601/16/15/2719/s1, Table S1: Memo.
