# Supplementary material for: Patients’ and Caregivers’ Conceptualisations of Pressure Ulcers and the Process of Decision-Making in the Context of Home Care
Source: Int J Environ Res Public Health. 2019 Jul 30;16(15):2719. doi: 10.3390/ijerph16152719 (PMC6696391; doi:10.3390/ijerph16152719)
Supplement: Supplementary file 1 [file ijerph-16-02719-s001.pdf]

Supplementary Table S1

Memo

Personal motivation: When positive feelings associated with the implication in shared decision making, such a wellbeing, peace of mind, feelings of productivity and willingness to participate in the drafting of clinical practice guidelines appear in the participant`s speeches.

Facilitators for the process of shared decision-making in the home care of pressure ulcers

**Codes**	**Categories**	**Subcategories**
Involvement of primary care professionals	Closeness	
	Trust	
	The attitude of professionals	Effective communication
		The need to be listened to
Personal motivation	Positive feelings associated with the implication in decision making	Wellbeing
		Peace of mind
		Feelings of productivity
		Willingness to participate in the drafting of clinical practice guidelines
